# Sex Differences in Biological Markers of Health in the Study of Stress, Aging and Health in Russia

**DOI:** 10.1371/journal.pone.0131691

**Published:** 2015-06-29

**Authors:** Anna Oksuzyan, Maria Shkolnikova, James W. Vaupel, Kaare Christensen, Vladimir M. Shkolnikov

**Affiliations:** 1 Max Planck Institute for Demographic Research, Rostock, Germany; 2 Department of Epidemiology, Biostatistics, and Biodemography, University of Southern Denmark, Odense, Denmark; 3 Scientific and Clinical Institute of Pediatry at the Pirogov Moscow Medical University, Moscow, Russian Federation; 4 Max-Planck Odense Center on the Biodemography of Aging, Odense, Denmark; 5 Department of Clinical Genetics, Odense University Hospital, Odense, Denmark; 6 Department of Clinical Biochemistry and Pharmacology, Odense University Hospital, Odense, Denmark; 7 New Economic School, Moscow, Russian Federation; University of Catanzaro Magna Graecia, ITALY

## Abstract

**Background:**

The apparent contradiction that women live longer but have worse health than men, the so called male-female health-survival paradox, is very pronounced in Russia. The present study investigates whether men in Moscow are healthier than women at the level of biomarkers, and whether the associations between biomarkers and subjective health have sex-specific patterns.

**Materials:**

Previously collected data in the study of Stress, Aging, and Health in Russia (SAHR, n = 1800) were used to examine sex differences in biomarkers and their associations with physical functioning and self-rated health.

**Results:**

The present study found mixed directions and magnitudes for sex differences in biomarkers. Women were significantly disadvantaged with regard to obesity and waist circumference, whereas men had a tendency toward higher prevalence of electrocardiographic abnormalities. No sex differences were indicated in the prevalence of immunological biomarkers, and mixed patterns were found for lipid profiles. Many biomarkers were associated with physical functioning and general health. Obesity and waist circumference were related to lower physical functioning among females only, while major Q-wave abnormalities with high probabilities of myocardial infarction and atrial fibrillation or atrial flutter were associated with physical functioning and self-rated health among males only.

**Conclusion:**

No clear patterns of sex differences in prevalence of high-risk levels of biomarkers suggest that the male-female health-survival paradox is weaker at the level of health biomarkers. We found some evidence that certain biomarkers reflecting pathophysiological changes in the organism that do not possess acute health risks, but over many years may lead to physical disability, are associated with physical functioning and self-rated health in women, whereas others reflecting more serious life-threatening pathophysiological changes are associated with physical functioning and self-rated health in men.

## Introduction

Much of the literature has shown that women have higher survival rates but worse health compared with men of the same age, so called male-female health-survival paradox [1–3]. The magnitude of the male-female gap in life expectancy has been found to be smaller in western European (EU) countries than in the eastern EU countries [4]. Research evidence suggests that despite lower mortality at all ages, women experience worse health than do men. International comparison studies involving European countries, US, and Japan show consistent male advantage in handgrip strength, reported physical functioning, and levels of depression symptomatology compared with their female counterparts [5–7]. A number of studies found that women report worse general health, although others revealed only small gender differences in self-reported health [8–11]. Epidemiological evidence suggests that the direction and magnitude of sex differences in health are mixed and differ across various health measures, geographic settings and ages under investigation.

The picture is more complex with regard to sex differences in the distributions of chronic conditions and biological markers of health [5, 12]. There is extensive research literature showing that the rates of coronary heart disease (CHD) and stroke are substantially lower in women, who also develop CHD about 10 years later than men [13–15]. However, after menopause the male-female ratio of incidence rates of acute myocardial infarction (MI) and stroke diminishes, and after age 75 the sex gap is very small [15] or is even reversed for the stroke incidence rates [13, 14].

A wealth of data on blood pressure, diabetes mellitus and impaired glucose tolerance, lipid profiles, excess weight and obesity also showed varying patterns of sex differences. Some studies reported a male disadvantage in the prevalence of diastolic hypertension and isolated systolic hypertension until about age 50 and, in the incidence and prevalence rates of diabetes mellitus, and impaired glucose tolerance, whereas others found no gender differences in the levels of diastolic blood pressure (DBP) in normotensive populations [16–18] and in the prevalence of physician-diagnosed or reported diabetes [5, 19]. The female disadvantage was demonstrated for hypertension after the fifth decade of life, lipoprotein levels, obesity and waist circumference [20–23]. Inconsistencies were indicated even within the same group of biomarkers: total cholesterol, low density lipoprotein (LDL), and high density lipoprotein (HDL) levels were found to be higher in women than in men at all ages, whereas triglycerides concentrations were similar in both sexes [24].

The epidemiological evidence on sex differences in inflammation markers is incoherent as well. Some studies show that C-reactive protein (CRP) and fibrinogen concentrations were higher among women than among men of the same age [25, 26], whereas others reported no gender differences in CRP levels [27] or even higher concentrations in men [28].

Previous research on the prevalence of electrocardiographic (ECG) abnormalities in the general population suggests that sex differences occur in some ECG findings. Many studies have demonstrated that left ventricular hypertrophy, atrial fibrillation and bundle branch blocks are more prevalent among men, and that ST-T abnormalities and ischemia-like ECG changes are more prevalent among women, whereas the prevalence of minor ECG changes are similar in both sexes [29–32].

Apparently, the direction and magnitude of sex differences in chronic conditions and biomarkers of health depends on a selected indicator and its definition (e.g. for hypertension or metabolic syndrome), the age of the study populations, and the data collection mode (self-reports or physician-diagnosed). It should be noted that many studies were conducted among adults younger than 75 years, whereas sex differences in biomarkers levels and prevalence of high-risk levels of biomarkers may change with +advanced age. Moreover, most studies were conducted in the US, western European countries, and Japan. There is less epidemiological evidence about sex-specific distributions of chronic diseases and biomarkers in Russia or other countries of the former Soviet Union.

The male-female health-survival paradox is very pronounced in Russia. Researchers have demonstrated that Russian males have exceptional mortality excess compared to Russian females [33, 34], but still they report better health and functioning [35, 36]. In 2013, the female-male gap in life expectancy was 11.2 years (76.3 and 65.1 years for Russian women and men, respectively), making it one of the highest in the world [37]. Less is known about the sex differences in various health measures in Russia. A recent study on the metabolic syndrome has demonstrated that Muscovite women experience a much higher prevalence of obesity, whereas men experience a much higher incidence of MI, a higher prevalence of hypertension and of hyperglycemia [38]. It has also been reported that Russian men have substantially better general and psychological health, but they have considerably higher levels of smoking and alcohol consumption and a higher incidence of related cancers than Russian women [38–41].

Previous research has suggested that men face more severe forms of health conditions or more fatal chronic diseases, whereas women suffer from less fatal but more disabling chronic conditions [12, 42]. This sex-specific distribution of chronic conditions may play an important role in explaining a strong male-female health-survival paradox in Russia. It is also possible that health advantage of Russian men compared with Russian women is less apparent at the level of objective health measures. Therefore, the questions investigated in the present study are whether men also maintain this advantage “under the skin”, i.e. when biomarkers of health are considered, and whether the associations between biological markers and subjective health measures are stronger in men than in women. We hypothesize that certain biomarkers reflecting pathophysiological changes in the organism that do not possess acute health risks, but over many years may lead to physical disability, would be more prevalent and more strongly associated with physical functioning and self-rated health in women than in men, whereas others reflecting more acute life-threatening pathophysiological changes in the body would be more prevalent and more strongly associated with physical functioning and general health in men than in women

## Materials and Methods

### Study population

The present investigation was based on the study of Stress, Aging, and Health in Russia (SAHR), a prospective population-based cohort study of the Moscow population aged 55 and older [43]. The study was conducted jointly by the State Research Center for Preventive Medicine (Moscow, Russian Federation), the Max Planck Institute for Demographic Research (Rostock, Germany) and Duke University (Durham, USA). The SAHR study participants were randomly selected from seven epidemiological cohorts, the Lipid Research Clinics (LRC) and MONICA cohorts, designed in the mid-1970s–1990s. Because the epidemiological cohorts included the residents of Moscow before the mid-1980s, additional participants representing those who moved to Moscow after 1985 were identified from the Moscow Outpatient Clinics’ registry. The SAHR baseline survey was conducted between December 2006 and June 2009 and included 1800 participants. The final response rate was 64%. Face-to-face interviews and extensive medical examinations were usually administered at the hospital; only participants unable or reluctant to come to the hospital were interviewed in their own homes, using the hospital protocol. The study involves a secondary data analysis of existing survey data. The SAHR data collection was approved by the Ethical Committee of the State Research Center for Preventive Medicine, Moscow, Russia and the Institutional Review Board at Duke University, Durham, USA. Written informed consent was obtained from participants to collect all data, including biological (grip strength, blood sample, urine sample, and Holter), and to use respective information for scientific purposes. All participant information was anonymized and de-identified prior to analyses.

### Health outcomes and biological markers of health

In the SAHR, the question about global self-rated health was a part of the Short Form Health Survey (SF-36) [44, 45]. In order to investigate sex differences in the prevalence of poor general health and its association with biomarkers, the response options excellent, very good, good, and fair/acceptable were combined into the higher category, whereas the responses poor and very poor were collapsed into the lower category.

Self-reported physical functioning in the SAHR was assessed using 10 items from the Physical Function section of SF-36 [44, 46]. The participants were asked to evaluate how much their health limits the performance of various activities on a usual day, ranging from bathing or dressing to moderate and vigorous activities, such as moving a table, running, lifting heavy objects, etc. There were three response options that reflect the presence and the degree of physical limitations: 1—yes, limited a lot, 2—yes, limited a little, 3—no, not limited. It has been shown that SF-36 physical function scores can be used as a valid measure of mobility disability in epidemiological studies in old-aged populations [47]. A standard procedure was used to calculate physical functioning score ranging from 0, indicating complete disability, to 100, indicating full functioning [44, 46]. As the physical functioning score was negatively skewed, for the present analysis it was recoded into a dichotomous outcome with poor physical functioning being the lowest quintile (0–55 in women, 0–60 in men) vs. all others (56–100 in women, 61–100 in men). To evaluate the history of MI, stroke and heart failure, participants were asked whether they have been ever told by a doctor whether they have had or have now any of these diseases (response options ‘have had’ and ‘have now’).

Smoking status was defined as never vs. current or former smoker. Reported frequency of alcohol consumption over the past 12 months was coded as never, once or twice a week, or more than twice a week. The presence of alcohol related problems in the past was assessed using three questions: history of drinking too much, history of being arrested for being drunk, and history of medical treatment of alcohol problems. Participants answering positively to any of the three questions were considered as having alcohol related problems in the past.

The biological markers of health were taken from clinical data that included anthropometric measurements, measurements of blood pressure, resting heart rate, a fasting blood specimen, and a 12-lead standard ECG in a supine position. Anthropometry included body height measured with a wall-mounted stadiometer, body weight measured with calibrated scales, waist and hip circumferences measured with a calibrated tape in the standing position [43]. The standard cutoffs were used to define low versus high-risk levels of total cholesterol, high density lipoproteins (HDL), triglycerides [48], obesity [49], waist circumference [50], Glycosylated hemoglobin (Hb) [51] and C-reactive protein (CRP)[52] that had been previously used with the SAHR data (Table 1) [53]. For a few other biomarkers, the high-risk group was defined as the highest quintile of unweighted sex-specific distribution for interleukin-6 (IL-6) and fibrinogen vs. all others (Table 1). Hypertension grade 1 was defined as systolic blood pressure (SBP) > = 140 and/or diastolic blood pressure (DBP) > = 90 mmHg and/or anti-hypertensive drug use, and hypertension grade 2 was defined as SBP > = 160 and/or DBP > = 100 mmHg and/or anti-hypertensive drug use [54]. ECG findings were coded using the Minnesota Classification of Electrocardiographic Findings [55]. Major Q-wave abnormalities with a high probability of MI (major Q-wave abnormalities) included 1-1-1 to 1-1-7 and 1-2-1 to 1-2-7 codes, atrial fibrillation or atrial flutter (AF) included 8-3-1 to 8-3-4 codes, and left ventricular hypertrophy plus ST-T abnormalities (LVH-ST abnormalities) included 3–1 and 3–3 codes.

**Table 1:** Cutoff values defining high-risk levels of biomarkers.

Biomarker	Cutoff level
Total cholesterol^a^	> = 6.216 mmol/l
High density lipoproteins^a^	<1.036 mmol/l
Triglycerides^a^	> = 2.26 mmol/l
Obesity^b^	> = 30.0
Waist circumference^c^	>102 cm men, >88 cm women
Hypertension Grade 1^d^	Systolic blood pressure > = 140 mm Hg or diastolic blood pressure > = 90 mmHg or anti-hypertensive drug use
Hypertension Grade 2^d^	Systolic blood pressure > = 160 mm Hg or diastolic blood pressure > = 100 mmHg or anti-hypertensive drug use
Glycosylated hemoglobin^e^	>6.5%
C-reactive protein^f^	> 3 mg/dl
Interleukin-6^g^	> = 2.16 pg/mL men, > = 1.84 mg/dl women
Fibrinogen^g^	> = 4.43 g/L men, > = 4.42 g/L women
Major Q-wave with a high probability of myocardial infarction^h^	Minnesota codes: 1-1-1 to 1-1-7 and 1-2-1 to 1-2-7
Atrial fibrillation or atrial flutter^h^	Minnesota codes: 8-3-1 to 8-3-4
Left ventricular hypertension with ST-T segment abnormalities^h^	Minnesota codes 3–1 and 3–3

a) Expert Panel on Detection Evaluation and Treatment of High Blood Cholesterol in Adults. Executive summary of the Third Report of the National Cholesterol Education Program (NCEP) Expert Panel on Detection, Evaluation, and Treatment of High Blood Cholesterol in Adults (Adult Treatment Panel III). JAMA 2001; 285: 2486–2497.

b) NHLBI Obesity Education Initiative Expert Panel on the Identification, Evaluation, and Treatment of Obesity in Adults (US). Clinical guidelines on the identification, evaluation, and treatment of overweight and obesity in adults—the evidence report. Obes Res 1998;6:51S-209S.

c) WHO. Waist circumference and waist-hip ratio: Report of a WHO expert consultation, Geneva 2011: World Health Organization.

d) Mancia G, Fagard R, Narkiewicz K, et al. ESH/ESC Guidelines for the management of arterial hypertension: The Task Force for the management of arterial hypertension of the European Society of Hypertension (ESH) and of the European Society of Cardiology (ESC). Eur Heart J 2013;34:2159–2219.

e) Rodbard H, Blonde L, Braithwaite S, Brett E, Cobin R, et al. American Association of Clinical Endocrinologists Medical Guidelines for Clinical Practice for the Management of Diabetes Mellitus. Endocrine Practice 2007; 13: 1–68.

f) Pearson TA, Mensah GA, Alexander RW, et al. Markers of Inflammation and Cardiovascular Disease: Application to Clinical and Public Health Practice: A Statement for Healthcare Professionals From the Centers for Disease Control and Prevention and the American Heart Association. Circulation 2003;107:499–511.

g) Defined by the highest quintile of the sex-specific distribution.

h) Prineas RJ, Crow RS, Zhang Z-M. The Minnesota Code Manual of Electrocardiographic Findings. London: Springer-Verlag London New York; 2010.

### Statistical analysis

A Chi-square test was used to examine sex differences in the prevalence of poor self-rated health and physical functioning and high-risk levels of biomarkers. To facilitate the comparison of poor self-rated health, physical functioning, and high-risk biomarker levels, especially those with low occurrences, the prevalence were standardized with respect to age by the direct method using the European population standard [56].

Logistic regression analysis was used to examine the association of poor physical functioning and self-rated health with biomarkers. To observe to what extent the inclusion of a biomarker attenuates the effect of sex, we initially run the basic model with sex, age and quadratic age only (Model 1). Model 2 examines the associations between health outcomes and each biomarker separately adjusted for sex, age, quadratic age, smoking status and current frequency of alcohol consumption and past alcohol problems. Similar analyses were carried out in sex-specific samples. Since the number of women with past alcohol problems was very small, this variable was omitted from the model in the female sample. To examine whether the associations between health outcomes and biomarkers are significantly different in men and women, additional analyses were conducted including the interactions between sex and the biomarkers into Model 2 (results not shown). All data analyses were performed using Intercooled Stata 13 [57].

The study involves a secondary data analysis of existing survey data. The SAHR data collection was approved by the Ethical Committee of the State Research Centre for Preventive Medicine, Moscow, Russia and the Institutional Review Board at Duke University, Durham, USA.

## Results

### Sex differences in the high-risk levels of biomarkers

In total, 961 (53.4%) women with a mean age of 67.7 years (standard error [SE] = 0.24, range: 55–92) and 839 (46.6%) men with a mean age of 68.9 years (SE = 0.28, range: 55–91) participated in the SAHR. The percentage of missing values on biomarkers was less than 1% and no substitution of missing values was made.

Women had substantially higher age-specific and age-standardized prevalence of poor physical functioning and poor self-rated health (Table 2). Statistically significant sex differences were indicated in the prevalence of former or current smoking (Table 3) and drinking alcohol more than twice a week (24.5% men vs. 3.9% women, p = 0.008). However, the proportions of never (2.7% in men vs. 6.5% in women) and moderate (72.7% in men vs. 89.5%, in women) drinking were similar. Muscovite men reported substantially more of alcohol related problems in the past than Muscovite women (n = 174, 21.6% vs. n = 10, 1.21%, respectively, p<0.001).

**Table 2:** Descriptive statistics of poor physical functioning and poor self-rated health.

	Men			Women			
Age	No	%	SE^a^	No	%	SE	p-value^b^
**Poor physical functioning**							
55–64	284	5.63	1.37	345	9.57	1.58	>0.05
65–74	319	12.54	1.85	445	22.92	1.99	<0.001
75+	229	34.06	3.13	168	47.62	3.85	0.006
Total	832	16.11	1.27	958	22.44	1.35	0.001
ASP	832	13.00	1.18	958	20.73	1.35	<0.001
**Poor self-rated health**							
55–64	285	10.18	1.79	346	16.18	1.98	0.028
65–74	322	11.49	1.78	447	20.36	1.90	0.001
75+	229	26.64	2.92	168	39.88	3.78	0.005
Total	836	15.19	1.24	961	22.27	1.34	<0.001
ASP	836	13.59	1.18	961	21.82	1.35	<0.001

a: SE—standard error, ASP–age–standardized prevalence

b: p–value for sex difference in the prevalence of poor physical functioning and poor self-rated health

**Table 3:** Prevalence of high-risk levels of biomarkers.

	Men			Women			
Age	No	%	SE	No	%	SE	p-value
**Behavioral factors**							
**Smoking**							
Total	837	66.19	1.64	961	19.46	1.28	<0.001
ASP^a^	837	68.35	1.65	961	22.04	1.42	<0.001
**Past alcohol problems**							
Total	804	21.64	1.45	826	1.21	0.38	<0.001
ASP	804	23.96	1.62	826	1.49	0.47	<0.001
**Alcohol consumption >3 times/week**							
Total	741	24.56	1.58	831	3.97	0.68	<0.001
ASP	741	25.25	1.69	831	4.03	0.71	<0.001
**Cardiovascular and metabolic**							
**Total cholesterol**							
Total	839	27.77	1.55	958	49.58	1.62	<0.001
ASP	839	29.25	1.67	958	49.41	1.69	<0.001
**HDL**							
Total	838	31.03	1.60	957	15.67	1.18	<0.001
ASP	838	31.61	1.70	957	16.67	1.29	<0.0001
**Triglycerides**							
Total	839	9.30	1.00	958	8.56	0.90	>0.05
ASP	839	10.22	1.14	958	9.31	1.01	>0.05
**Obesity**							
Total	838	27.09	1.54	959	43.48	1.60	<0.001
ASP	838	28.92	1.67	959	43.99	1.68	<0.001
**Waist circumference**							
Total	835	32.34	1.62	959	58.08	1.59	<0.001
ASP	835	32.42	1.71	959	59.44	1.65	<0.001
**Hypertension grade 1**							
Total	839	54.11	1.72	961	55.25	1.60	>0.05
ASP	839	53.50	1.82	961	53.57	1.67	>0.05
**Hypertension grade 2**							
Total	839	70.68	1.57	961	68.26	1.50	>0.05
ASP	839	69.11	1.70	961	66.80	1.59	>0.05
**Glycosylated Hb**							
Total	832	15.75	1.26	953	16.79	1.21	>0.05
ASP	832	15.53	1.32	953	16.71	1.27	>0.05
**Low grade inflammation**							
**CRP**							
Total	830	28.92	1.57	947	28.93	1.47	>0.05
ASP	830	28.77	1.65	947	29.10	1.55	>0.05
**IL-6**							
Total	833	20.17	1.39	955	20.21	1.30	>0.05
ASP	833	19.50	1.43	955	20.62	1.36	>0.05
**Fibrinogen**							
Total	836	20.45	1.40	957	20.17	1.30	>0.05
ASP	836	20.30	1.47	957	20.10	1.36	>0.05
**Electrocardiographic**							
**Major Q-wave**							
Total	839	6.67	0.86	961	4.27	0.65	<0.05
ASP	839	6.42	0.88	961	4.13	0.66	<0.05
**AF**							
Total	839	5.24	0.77	961	3.43	0.59	>0.05
ASP	839	4.42	0.68	961	3.16	0.55	>0.05
**LVH-ST**							
Total	839	3.34	0.62	961	1.35	0.37	<0.01
ASP	839	3.18	0.62	961	1.33	0.38	<0.01
**Reported diseases**							
**Stroke**							
Total	835	9.70	1.02	959	6.05	0.77	<0.01
ASP	835	8.54	0.96	959	5.54	0.73	<0.05
**MI**							
Total	835	14.73	1.23	960	5.31	0.72	<0.001
ASP	835	12.98	1.15	960	5.22	0.73	<0.001
**Heart failure**							
Total	834	21.46	1.42	959	27.32	1.44	<0.01
ASP	834	18.35	1.29	959	26.13	1.40	<0.001

a: ASP–age-standardized prevalence (based on the standard European population), SE–standard error, HDL—high density lipoproteins, Hb–hemoglobin, CRP—C-reactive protein, IL-6—interleukin-6, MI–myocardial infarction, Major Q-wave–Major Q-wave abnormalities with high MI probability, AF–atrial fibrillation or atrial flutter, LVH-ST—Left ventricular hypertrophy plus ST-T abnormalities.

Women were significantly disadvantaged with regard to total cholesterol, obesity and waist circumference, but they had favorable prevalence of HDL levels compared with men at all ages. No sex differences were found in the age-standardized prevalence (ASP) of high-risk levels of triglycerides, glycosylated Hb and all three inflammation markers, CRP, IL-6, and fibrinogen. There was a tendency toward higher prevalence of ECG abnormalities among men than among women, with significant sex differences in the ASP of major Q-wave abnormalities with a high probability of MI and LVH-ST abnormalities. Men reported a history of MI and stroke more often than did their female counterparts at all ages, but the prevalence of heart failure was sigificantly higher in women and no sex differences were found in the ASP of grade 1 and 2 hypertension.

### Association of biomarkers with physical functioning

All associations were in the expected directions where women and persons with high-risk levels of biomarkers were at higher risks of having low physical functioning (Table 4). The latter was significantly related to high-risk levels of HDL, triglycerides, obesity, waist circumference, CRP, IL-6, and fibrinogen in the total sample. No ECG variables were significantly associated with physical functioning. Those individuals with history of stroke, MI, and heart failure were also at significantly higher risks of haivng lower physical functioning.

**Table 4:** Association of physical functioning with biomarker levels in total and sex-specific samples.

	TOTAL SAMPLE			MEN			WOMEN		
	OR	SE	p-value	OR	SE	p-value	OR	SE	p-value
**Model 1** ^**a**^									
Women	1.88	0.25	<0.001						
**Model 2**									
**Cardiovascular and metabolic**									
**Total cholesterol**	0.98	0.15	>0.05	0.98	0.25	>0.05	1.07	0.19	>0.05
Women	2.04	0.37	<0.001						
**HDL** ^**b**^	1.56	0.26	0.008	1.80	0.42	0.011	1.43	0.35	>0.05
Women	2.15	0.39	<0.001						
**Triglycerides**	1.71	0.42	0.028	1.36	0.52	>0.05	2.23	0.70	0.010
Women	2.04	0.36	<0.001						
**Obesity**	1.70	0.25	<0.001	1.43	0.35	>0.05	1.91	0.35	<0.001
Women	1.87	0.34	0.001						
**Waist circumference**	1.67	0.25	<0.001	1.32	0.30	>0.05	1.88	0.36	0.001
Women	1.74	0.32	0.002						
**Hypertension gr. 1**	1.24	0.20	>0.05	1.03	0.25	>0.05	1.38	0.28	>0.05
Women	2.03	0.36	<0.001						
**Hypertension gr. 2**	1.38	0.20	0.027	1.40	0.31	>0.05	1.37	0.25	>0.05
Women	2.01	0.36	<0.001						
**Glycosylated Hb**	1.20	0.22	>0.05	1.08	0.31	>0.05	1.31	0.31	>0.05
Women	2.11	0.38	<0.001						
**Low grade inflammation**									
**CRP**	1.81	0.27	<0.001	1.80	0.43	0.012	1.68	0.32	0.007
Women	1.90	0.35	<0.001						
**IL-6**	1.56	0.26	0.007	2.17	0.53	0.002	1.17	0.26	>0.05
Women	1.97	0.35	<0.001						
**Fibrinogen**	1.79	0.30	<0.001	2.22	0.55	0.001	1.52	0.33	0.052
Women	1.96	0.35	<0.001						
**Electrocardiographic**									
**Major Q-wave**	1.31	0.39	>0.05	2.98	1.13	0.004	0.50	0.26	>0.05
Women	2.04	0.36	<0.001						
**AF**	1.20	0.40	>0.05	0.74	0.36	>0.05	1.87	0.88	>0.05
Women	2.03	0.36	<0.001						
**LVH–ST**	1.39	0.62	>0.05	0.74	0.49	>0.05	3.26	2.25	>0.05
Women	2.05	0.37	<0.001						
**Reported diseases**									
**Stroke**	2.21	0.53	0.001	2.02	0.66	0.033	2.54	0.87	0.006
Women	2.06	0.37	<0.001						
**MI**	2.01	0.46	0.002	2.04	0.57	0.010	1.63	0.62	>0.05
Women	2.12	0.38	<0.001						
**Heart failure**	2.78	0.42	<0.001	3.18	0.77	<0.001	2.75	0.52	<0.001
Women	1.83	0.33	0.001						

a: Model 1 includes sex, age, and quadratic age; Model 2: Model 1 plus biomarker (each separately), smoking status, frequency of current alcohol consumption, and presence of alcohol problems in the past

b: HDL—high density lipoproteins, Hb–hemoglobin, CRP—C-reactive protein, IL-6—interleukin-6, MI–myocardial infarction, Major Q-wave–Major Q-wave abnormalities with high MI probability, AF–atrial fibrillation or atrial flutter, LVH-ST—Left ventricular hypertrophy plus ST-T abnormalities, OR–odds ratio, CI–confidence interval.

A similar analysis in sex-specific strata showed that reported stroke and heart failure were significantly associated with physical functioning in both sex-specific samples. High-risk levels of triglycerides, obesity, and waist circumference were significantly associated with physical functioning in the female sample only. HDL, IL-6, fibrinogen, and major Q-wave abnormalities were significantly associated with physical functioning in the male sample. None of reported diseases was associated with physical functioning among women, but history of MI was positively related to poor physical functioning among men. The analysis of sex-specific patterns of the relationships between physical functioning and biomakers showed that only the interaction between sex and major Q-wave abnormalities was statistically significant (OR = 0.17, 95%CI: 0.05, 0.58, p-value = 0.005).

### Association of biomarkers with self-rated health

As with the analysis of the relationships between physical functioning and biomarkers, women and individuals with high-risk levels of biomarkers were at a higher risk of reporting poor self-rated health (Table 5). Higher levels of glycosylated Hb, CRP, IL-6, and fibrinogen, obesity, waist circumference, presence of hypertension grade 2, and AF were significantly related to poor general health in the total sample. The analysis of sex-specific patterns of the relationships between general health and biomakers showed that the interaction between sex and IL-6 (OR = 0.52, 95%CI = 0.28, 0.99, p-value = 0.047) and major Q-wave abnormalities (OR = 0.28, 95%CI = 0.08, 0.95, p-value = 0.041) were statistically significant.

**Table 5:** Association of poor self-rated health with biomarker levels in the total and sex-specific samples.

	TOTAL SAMPLE			MEN			WOMEN		
	OR	SE	p-value	OR	SE	p-value	OR	SE	p-value
**Model 1** ^**a**^									
Women	1.80	0.23	<0.001						
**Model 2**									
**Cardiovascular and metabolic**									
**Total cholesterol**	1.10	0.16	>0.05	1.21	0.29	>0.05	1.15	0.20	>0.05
Women	1.79	0.32	0.001						
**HDL** ^**b**^	1.33	0.23	>0.05	1.16	0.28	>0.05	1.47	0.35	>0.05
Women	1.88	0.34	<0.001						
**Triglycerides**	1.50	0.36	>0.05	1.39	0.52	>0.05	1.62	0.49	>0.05
Women	1.83	0.33	0.001						
**Obesity**	1.53	0.22	0.004	1.73	0.41	0.022	1.47	0.26	0.028
Women	1.71	0.31	0.003						
**Waist circumference**	1.37	0.20	0.031	1.19	0.27	>0.05	1.43	0.26	0.049
Women	1.68	0.31	0.004						
**Hypertension gr. 1**	1.15	0.18	>0.05	0.85	0.20	>0.05	1.34	0.27	>0.05
Women	1.84	0.33	0.001						
**Hypertension gr. 2**	1.39	0.20	0.023	1.29	0.29	>0.05	1.43	0.26	0.053
Women	1.83	0.33	0.001						
**Glycosylated Hb**	1.59	0.28	0.008	1.48	0.41	>0.05	1.59	0.36	0.038
Women	1.90	0.34	<0.001						
**Low grade inflammation**									
**CRP**	1.38	0.21	0.035	1.05	0.26	>0.05	1.54	0.29	0.020
Women	1.78	0.32	0.001						
**IL-6**	1.69	0.28	0.001	2.44	0.59	<0.001	1.15	0.25	>0.05
Women	1.78	0.32	0.001						
**Fibrinogen**	1.66	0.27	0.002	1.54	0.40	>0.05	1.80	0.37	0.004
Women	1.82	0.33	0.001						
**Electrocardiographic**									
**Major Q-wave**	1.28	0.38	>0.05	2.33	0.89	0.027	0.64	0.32	>0.05
Women	1.85	0.33	0.001						
**AF**	1.96	0.60	0.028	2.32	0.91	0.032	1.45	0.68	>0.05
Women	1.86	0.33	<0.001						
**LVH-ST**	1.66	0.71	>0.05	1.73	0.93	>0.05	1.67	1.12	>0.05
Women	1.87	0.33	<0.001						
**Reported diseases**									
**Stroke**	2.30	0.54	<0.001	2.83	0.88	0.001	1.58	0.55	>0.05
Women	1.86	0.33	0.001						
**MI**	1.93	0.44	0.004	2.11	0.59	0.007	1.73	0.63	>0.05
Women	1.92	0.34	<0.001						
**Heart failure**	3.09	0.47	<0.001	3.26	0.78	<0.001	3.31	0.63	<0.001
Women	1.66	0.30	0.005						

a: Model 1 includes sex, age, and quadratic age; Model 2: Model 1 plus biomarker (each separately), smoking status, current alcohol consumption, and presence of alcohol problems in the past.

b: HDL—high density lipoproteins, Hb–hemoglobin, CRP—C-reactive protein, IL-6—interleukin-6, MI–myocardial infarction, Major Q-wave–Major Q-wave abnormalities with high MI probability, AF–atrial fibrillation or atrial flutter, LVH-ST—Left ventricular hypertrophy plus ST-T abnormalities, OR–odds ratio, CI–confidence interval.

The analyses in sex-specific samples revealed that obesity was significantly associated with poor self-rated health in both men and women. High-risk levels of glycosylated Hb, waist circumference, CRP, fibrinogen, and hypertension grade 2 (marginally only) were significantly associated with increased risks of reporting poor general health in the female sample. In the male sample, high-risk levels of IL-6, major Q-wave abnormalities and AF were significantly associated with elevated risks of reporting poorer self-rated health. The history of stroke and MI was also positively significantly related to lower ratings of general health among men.

## Discussion

The present study found mixed directions and magnitudes of sex differences in biomarkers. Even within the same biomarker group different patterns were indicated: no sex difference in the prevalence of high-risk levels of triglycerides, male advantage with respect to total cholesterol and female advantage with respect to HDL cholesterol. Men and women had similar prevalence of high-risk levels of inflammation markers, glycosylated Hb, and hypertension grades 1 and 2. High-risk levels of inflammation markers and hypertension grade 2 were significantly related to lower physical functioning and poor self-rated health. Besides, our study suggested that some groups of biomarkers showed predominantly female or male advantage. In line with previous research findings, prevalence of obesity and high-risk levels of waist circumference were higher among female Muscovites than among their male counterparts [21, 22]. Furthermore, the anthropometric measurements were associated with physical functioning among women only. This suggests that excess weight may explain high disability levels at older ages among women in Moscow and also supports previous research that women suffer disproportionally more from excess weight and BMI change with regard to morbidity compared with men of the same age [58].

The present study showed that men tended to have substantially higher prevalence of major Q-wave abnormalities with a high probability of MI and LVH-ST abnormalities than women and that their presence was significantly associated with worse health among men but not among women. Previous studies also indicated higher prevalence of arrhythmias and conduction defects among men, and higher prevalence of ST-T abnormalities and ischemia-like ECG changes among women [29, 30]. Research evidence has shown that the presence of major ECG abnormalities is associated with lower quality of life and increased risks of cognitive decline and loss of independence in carrying out activities of daily living [59, 60]. However, no study focused on sex differences in the associations between ECG abnormalities and subjective health measures, and controversial reports exist with regard to sex-specific associations of ECG abnormalities with mortality risk. Some studies found no sex differences in predictive values of ECG abnormalities for all-cause and coronary heart disease mortality [61, 62], while others reported a higher relative mortality risk in men than in women [29, 63].

Prevalence of smoking was about three-fold higher among Muscovite men compared with women, but it was not associated with physical functioning and self-rated health in both sexes. This agrees with prior studies that showed that smoking was related to substantially lower probability of surviving from age 40 to 70 years (45% vs. 70%) in Russia, but it was unrelated to the rating of general health among adult Russians [39, 64]. It has been suggested that greater sex differences in lifestyle behavior may partially explain why the contradiction in sex differences in health and mortality is so strong in Russia.

Our findings with regard to sex differences in the prevalence of lipoprotein biomarkers agree with the Cardiovascular Health Study reports on higher concentrations of total and HDL cholesterol among women than among men and no sex differences in triglycerides, except for the oldest age group [24]. At first, it may appear surprising that in the present study very few lipid variables were associated with selected health outcomes, as an unfavorable lipid profile is a well-established risk factor of all-cause and cardiovascular mortality [65, 66]. However, prior research has shown that Russians have relatively low total and LDL cholesterol concentrations and relatively high HDL cholesterol levels, and that blood lipids are rather weakly linked to the risk of cardiovascular death [67, 68]. Although these inconsistent findings are not fully understood, the authors tended to attribute the high cardiovascular mortality in Russia to possible myocardial damage due to high alcohol intake and/or to unusual dietary differences across educational groups, where the least educated group had more favorable total and LDL cholesterol profiles and the lowest saturated fat intake. In addition, some international studies have found only weak associations of plasma lipids with cardiovascular deaths or have demonstrated that low levels of lipids are risk factors for health deterioration among old-aged persons [69, 70].

Generally, there is compelling evidence that the prevalence of hypertension is higher in men than in women at younger ages and that the sex gap is small around the sixth decade of life, reversing at advanced ages [23, 71]. It has been also well established that the MI incidence is higher among men than women throughout life [72, 73]. Similar sex-specific patterns have been reported for stroke, although some studies revealed no sex differences or higher rates in women than in men aged 85 years and older [74, 75]. Although limited evidence extists regarding sex-specific associations of hypertension, MI, stroke, and heart failure with reported health outcomes, a comprehensive review of sex differences in stroke epidemiology suggests that women generally have more physical impairments after stroke compared with their male counterparts [14].

Some data demonstrated that men have weaker immune responses than women, which is partially due to the immunosuppressive effect of testosterone [76–79]. Men’s greater susceptibility to infectious diseases is thought explain sex differences in health and mortality to some extent [80]. Previous research reports provided mixed findings suggesting similar levels of inflammatory markers in men and women [27, 81, 82], higher concentrations of respective markers among women [25, 26, 83–85] or among men [28].

The literature on sex differences in the levels of inflammatory markers and on the sex-specific effects of inflammatory markers on morbidity measures and mortality among old-aged individuals are limited and controversial. Studies of the Finnish and Danish populations have shown that inflammatory markers are associated with physical performance and physical functioning [86, 87], but no sex-specific associations were reported.

The present study demonstrates that the direction and magnitude of sex differences in health vary also across biomarkers of health. No clear pattern of sex differences in high-risk levels of biomarkers disagrees with our initial hypothesis that men are healthier not only with regard to physical performance and reported measures of health, but also in terms of biological markers of health. Nevertheless, no apparent male disadvantage in most biomarkers of health contradicts the substantially high male mortality in Moscow, which is about twice as high as that of women at ages 55–74 years. These findings suggest that the male-female health-survival paradox is weaker when biomarkers are considered as health measurements.

We found some evidence to support our initial hypothesis that certain biomarkers reflecting pathophysiological changes in the organism that do not possess acute health risks, but over many years may lead to physical disability would be more strongly associated with physical functioning and self-reported health in women than in men, whereas others reflecting more serious life-threatening pathophysiological changes in the heart would be more strongly associated with physical functioning and general health in men than in women. As such, obesity or waist circumference were associated with lower physical functioning among female Muscovites only, while major Q-wave abnormalities and AF were associated with physical functioning and self-rated health among Moscow men but not women. However, the lack of effect of major Q-wave abnormalities and AF on health outcomes in female Muscovites may be due to a small number of women diagnosed with these ECG changes.

The present study has several limitations. First, most biomarkers were measured only once and captured merely a snapshot of the complex dynamic processes in the human organism and are therefore subject to a measurement error. Second, the study utilized cross-sectional survey data that potentially inherit the selective nature of the study population and do not allow establishing the direction of the relationships between biomarkers and selected health outcomes. Third, the mortality selection in Russian men before age 55 is much stronger than in Russian women: probabilities of dying before age 55 in 2007–8 were 32% for Russian men and 12% for Russian women, and the respective probabilities of dying between ages 55 and 70 were 45% in men and 20% women [88]. Therefore, the selection of healthier men into the SAHR sample could increase sex differences in health and biomarkers in the sample. Forth, no post-stratification weights for age and education were used to bring the age-education composition of the sample to the general Russian population. However, analyses of sex differences in ageing trajectories in the same sample revealed similar patterns when post-stratification weights for age and education were used [results available upon request]. Fifth, logistic regression analyses included adjustments for age, quadratic age, smoking status, and alcohol while some known confounders were omitted. However, additional analyses with adjustment for education and marital status produced similar results and, thus, more parsimonious models were considered. Finally, specific laboratory measurements of biomarkers in the SAHR sample may result in different sex-specific patterns in the levels of biomarkers and, thus, the results may be not representative of other research settings.

In conclusion, the present study found no evidence that men in Moscow are healthier than their female counterparts with regard to biomarkers of health, suggesting that the male-female health-survival paradox is weaker when biological markers of health are considered. The present study found some support for the hypothesis that women’s apparently worse health and men’s substantially higher mortality can be partially explained by pathophysiological changes which progress slowly and may lead to disability over years or by pathophysiological changes that possess acute health risks, respectively. More studies in old-aged populations are needed to investigate the sex differences in biomarkers and the sex-specific associations of biomarkers with health outcomes.
